# Effects on quality of life of weekly docetaxel-based chemotherapy in patients with locally advanced or metastatic breast cancer: results of a single-centre randomized phase 3 trial

**DOI:** 10.1186/1471-2407-11-75

**Published:** 2011-02-16

**Authors:** Francesco Nuzzo, Alessandro Morabito, Adriano Gravina, Francesca Di Rella, Gabriella Landi, Carmen Pacilio, Vincenzo Labonia, Emanuela Rossi, Ermelinda De Maio, Maria Carmela Piccirillo, Giuseppe D'Aiuto, Renato Thomas, Massimo Rinaldo, Gerardo Botti, Maurizio Di Bonito, Massimo Di Maio, Ciro Gallo, Francesco Perrone, Andrea de Matteis

**Affiliations:** 1 National Cancer Institute, via M. Semmola, 80131 Napoli, Italy; 2 Department of Medicine and Public Health, Second University of Napoli, via L. Armanni, 80138 Napoli, Italy; 3 S. Giuseppe Moscati Hospital, Avellino; 4 Azienda USL 6 Livorno

## Abstract

**Background:**

To evaluate whether weekly schedules of docetaxel-based chemotherapy were superior to 3-weekly ones in terms of quality of life in locally advanced or metastatic breast cancer.

**Methods:**

Patients with locally advanced or metastatic breast cancer, aged ≤ 70 years, performance status 0-2, chemotherapy-naive for metastatic disease, were eligible. They were randomized to weekly or 3-weekly combination of docetaxel and epirubicin, if they were not treated with adjuvant anthracyclines, or docetaxel and capecitabine, if treated with adjuvant anthracyclines. Primary end-point was global quality of life change at 6-weeks, measured by EORTC QLQ-C30. With two-sided alpha 0.05 and 80% power for 35% effect size, 130 patients per arm were needed.

**Results:**

From February 2004 to March 2008, 139 patients were randomized, 70 to weekly and 69 to 3-weekly arm; 129 and 89 patients filled baseline and 6-week questionnaires, respectively. Global quality of life was better in the 3-weekly arm (p = 0.03); patients treated with weekly schedules presented a significantly worsening in role functioning and financial scores (p = 0.02 and p < 0.001). Neutropenia and stomatitis were worse in the 3-weekly arm, where two toxic deaths were observed. Overall response rate was 39.1% and 33.3% in 3-weekly and weekly arms; hazard ratio of progression was 1.29 (95% CI: 0.84-1.97) and hazard ratio of death was 1.38 (95% CI: 0.82-2.30) in the weekly arm.

**Conclusions:**

In this trial, the weekly schedules of docetaxel-based chemotherapy appear to be inferior to the 3-weekly one in terms of quality of life in patients with locally advanced or metastatic breast cancer.

**Trial registration:**

ClinicalTrials.gov NCT00540800.

## Background

Chemotherapy is a cornerstone of the treatment of advanced breast cancer. Taxanes represent, together with anthracyclines, a class of drugs with very strong evidence of efficacy. Particularly, docetaxel is effective both against locally advanced and metastatic breast cancer, either as single-agent [1-5] or in combination with other drugs, like anthracyclines [6-9] or capecitabine [10]. However, with such combinations, the most common side effects are myelo-suppression and its complications, neutropenic fever and/or infection. While docetaxel is usually given every 3 weeks, a weekly schedule was proposed to improve the toxicity profile of the drug, particularly neutropenia and febrile neutropenia, without decreasing antitumoral activity [11,12]. In metastatic breast cancer, weekly docetaxel as single agent was active and well tolerated, also in elderly patients and in those with deteriorated performance status [13,14]; similarly, combinations of weekly docetaxel with epirubicin or capecitabine are also characterized by a favourable toxicity profile and antitumor activity [15,16]. Particularly, weekly docetaxel and capecitabine have demonstrated preclinical antitumor synergy; this synergy is thought to occur from docetaxel-mediated up-regulation of thymidine phosphorylase, an enzyme responsible for the relative tumor selectivity of capecitabine [17]. However, no comparison in terms of quality of life between weekly and 3-weekly schedules of docetaxel-based chemotherapy has been reported to date.

On these bases, we planned a phase III randomized clinical trial to test whether a weekly schedule of docetaxel combined with either weekly epirubicin or capecitabine was superior to standard 3-weekly scheduling in terms of quality of life in patients with locally advanced or metastatic breast cancer.

## Methods

### Eligibility criteria

Women with locally advanced or metastatic breast cancer, aged ≤ 70 years, not previously treated with chemotherapy for metastatic disease were eligible. Patients were required to have an Eastern Cooperative Oncology Group performance status of 0 to 2, adequate bone marrow, (absolute granulocyte count ≥ 2,000/mmc, platelets ≥ 100,000/mmc, haemoglobin ≥ 10 g/dl), renal (serum creatinine ≤ 1.25 upper normal limit) and liver (total bilirubin ≤ 1.5 times upper normal limit; AST and ALT ≤ 1.25 upper normal limit in absence of liver metastases and ≤ 2.5 times upper normal limit in presence of liver metastases) function. Previous adjuvant or neoadjuvant chemotherapy as well as previous endocrine therapy for metastatic disease was allowed. Radiotherapy was allowed, either adjuvant or for metastatic disease. Patients were excluded if they had a positive history of other types of cancer (except for radically resected carcinoma-in-situ of the cervix or non-melanoma skin cancer), had received docetaxel as adjuvant chemotherapy, had symptomatic brain metastases, or serious medical conditions potentially compromising study participation. Pregnant or lactating women were ineligible. All patients were required to provide written informed consent; the Independent Ethical Committee of the National Cancer Institute of Naples approved the protocol (ClinicalTrials.gov NCT00540800).

### Treatment schedules

Patients not previously treated with anthracyclines were randomized to weekly or 3-weekly combination of docetaxel and epirubicin [15,18,19]; treatment schemes chosen for locally advanced breast cancer patients had a higher planned dose-intensity than those chosen for those with metastatic disease. Patients pre-treated with anthracyclines were randomized to weekly or 3-weekly combination of docetaxel plus capecitabine [16,20]. Dose and schedule details are reported in Table 1.

**Table 1:** Regimens and drug doses of control and experimental arms

Setting	Treatment Arm	Regimens	Dose	Schedule	Reference
**Locally advanced**	Control	Epirubicin	75 mg/m², d 1	Every 3 weeks for 4 cycles	de Matteis A, 2002
		Docetaxel	80 mg/m², d 1		
	Experimental	Epirubicin	30 mg/m², dd 1-8-15	Every 4 weeks for 3 cycles	Wenzel C, 2002
		Docetaxel	35 mg/m², dd 1-8-15		

**Metastatic, no previous anthracyclines**	Control	Epirubicin	60 mg/m², d 1	Every 3 weeks for 6 cycles	Trudeau ME, 1999
		Docetaxel	75 mg/m², d 1		
	Experimental	Epirubicin	25 mg/m², dd 1-8-15	Every 4 weeks for 6 cycles	Wenzel C, 2002
		Docetaxel	30 mg/m², dd 1-8-15		

**Metastatic, previously treated with anthracyclines**	Control	Capecitabine	1000 mg/m², b.i.d., dd 1-14	Every 3 weeks for 6 cycles	Pronk LC, 2000
		Docetaxel	75 mg/m², d 1		
	Experimental	Capecitabine	625 mg/m², b.i.d., dd 5-18	Every 4 weeks for 6 cycles	Nadella P, 2002
		Docetaxel	35 mg/m², dd 1-8-15		

Docetaxel was administered as 1-hour intravenous infusion. Epirubicin was administered as 10 minutes intravenous infusion, before docetaxel. Capecitabine was administered orally. Treatment delays for a maximum of 3 weeks or dose reduction of chemotherapy of 25% or 50% following hematological or non-hematological toxicity were planned by protocol.

Premedication with orally prednisone, 50 mg in 3-weekly and 25 mg in weekly arm, was performed as follows: at -12, -3 and -1 hours before chemotherapy and at +12, +24 and +36 hours after every administration of chemotherapy. Prophylactic antiemetic treatment with 5-HT3 antagonists was given from the first infusion. Granulocyte colony-stimulating factor (G-CSF) was administered at 5 mcg/kg/day subcutaneously in case of grade 4 neutropenia until neutrophil count >2000/mm^3^. Prophylactic use of G-CSF was mandated for patients with locally advanced breast cancer treated with 3-weekly docetaxel and epirubicin.

### Baseline and treatment evaluations

Staging included history and physical examination, performance status score, routine laboratory studies, ECG, chest radiographs, abdomen ultrasound scan or computed tomography, bone scan, skeletal radiographs (if the bone scan was abnormal). Patients were monitored weekly by complete hematology and before every cycle with complete chemistry analyses, record of toxicity, adverse events, and physical examination. Evaluation of tumor response was performed after 3 and 6 cycles for patients with metastatic disease: patients with major response or stable disease continued treatment up to a maximum of 6 courses. For patients with locally advanced disease evaluation of tumor response was performed after 4 cycles and thereafter surgery was planned, if feasible.

### Assessment of quality of life

Two instruments were applied: the European Organization for Research and Treatment of Cancer Quality of Life Core Questionnaire (EORTC QLQ-C30) and the Daily Diary Card. The EORTC QLQ-C30 is a 30-item self-reporting questionnaire [21]. It is composed of 5 multi-item functional subscales: physical, role, emotional, social and cognitive functioning; three multi-item symptom scales measuring fatigue, pain, and emesis; a global health status subscale; and six single items to assess financial impact and symptoms such as dyspnoea, sleep disturbance, appetite, diarrhea, and constipation. Scores were computed according to EORTC instructions [22]. For functioning scales (i.e. those exploring physical, role, emotional, cognitive and social functioning and global health status), the higher the value the better the level of function. For symptoms scales and items, the higher the value the worse the severity of symptoms. Questionnaires were administered before randomization and 6 weeks after beginning of treatment in both arms.

The Daily Diary Card was designed by the Medical Research Council Lung Cancer Working Party [23] to capture rapid and transient changes in health and quality of life, which may occur on a day-to-day basis during cancer treatment. Patients had to score 8 items (sleeping, mood, well-being, level of activity, nausea, vomiting, appetite loss and pain) daily, from day 1 (first day of chemotherapy administration) to day 42 (first 6 weeks of treatment).

### Assessment of objective response and toxicity

Response evaluation was performed according to response evaluation criteria in solid tumor (RECIST). Patients who stopped treatment because of death, toxicity or refusal before restaging were defined as non-responders in the calculation of response rate.

Toxicity was evaluated according to National Cancer Institute - Common Toxicity Criteria (NCIC-CTC) version 2.0. For each type of toxicity, the worst degree experienced throughout the treatment was computed for each patient.

### Calculation of dose-intensity

Total delivered dose (mg/m^2^) was calculated by summing all delivered drug doses. Actual time on treatment was calculated as the difference between the last date of treatment administration and the date of treatment start, considering the last cycle as lasting the planned number of weeks. Dose intensity was calculated dividing total dose by time on treatment and was expressed as mg/m^2^/week.

### Study design and sample size

The study was designed as a single centre randomised phase III study with quality of life as primary end-point. Global health status scale of EORTC QLQ-C30 after six weeks from the start of chemotherapy was used to plan sample size.

A sample size of 130 in each group was required to have 80% power to detect an effect size of 0,35 (i.e. a difference between mean scores of global health status equal to 35% of the standard deviation) after six weeks of chemotherapy, using a two group t-test with a 0,050 two-sided significance level (nQuery Advisor^® ^4.0, Statistical Solutions Ltd, Cork, Ireland). This difference, although small, was considered of potential clinical interest. Considering a small dropout rate, 280 overall patients were planned.

Secondary endpoints included response rate, toxicity and overall survival. Patients were randomly assigned to the two arms of the trial in a 1:1 ratio. Randomization was performed by means of a computer-driven minimization procedure. Stratification factors were: group of treatment (locally advanced vs metastatic not pretreated with anthracyclines vs metastatic previously treated with anthracyclines), performance status (0 vs. 1 vs. 2) and age (≤50, >50 years).

### Statistical analysis

Since the study was stopped prematurely, analyses of efficacy were definitely underpowered. For quality of life, the primary end-point of the study, the actual size gives an 80% power to detect an effect size of 48% for the experimental arm with a two-sided significance level of 0.05. At 6 weeks, mean differences from baseline values were calculated for each domain or symptom, within the two treatment groups. Differences from baseline scores were compared between treatments by a multivariable linear regression model, with treatment and baseline values as covariates. For Daily Diary Card, the daily rate of patients falling into the two worst scores was calculated for each item.

For secondary end-points only descriptive analyses were performed. Response rate was defined as the number of complete plus partial responses divided by the total number of patients enrolled in each comparison arm. Median follow-up was calculated according to the inverted Kaplan-Meier technique [24]. Progression-free survival was defined as the interval between the date of randomization and disease progression or death whichever occurred first; patients alive and not progressive were censored at the date of the last follow-up information. Overall survival was defined as the interval from the date of randomization and the date of death or the date of last follow-up information for living patients. For both progression-free and overall survival, median values and hazard ratios (HR) of weekly vs 3-weekly arms were reported.

Data are presented for the whole series of patients and scattered by the three subgroups defined by stage of disease and previous treatment with anthracyclines.

## Results

### Patient characteristics

From February 2004 to March 2008, 139 patients were enrolled in the study, 70 in the weekly and 69 in the 3-weekly arm. The enrollment was slower than planned and was definitively stopped following the publication of the results of a trial that compared weekly vs 3-weekly schedules of taxanes in adjuvant breast cancer, indicating that in adult patients docetaxel was more effective if given every 3 weeks [25].

**Figure 1:**
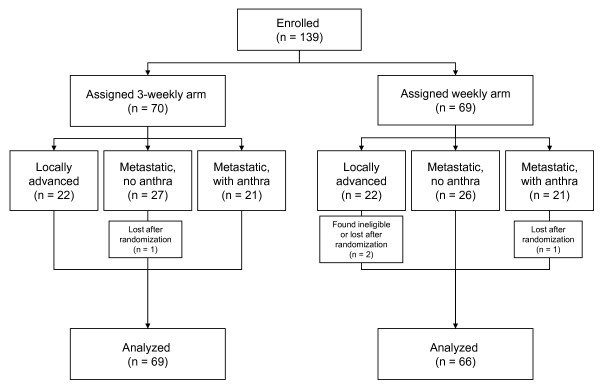
**The CONSORT flowchart**.

Two patients were lost, immediately after randomization, in the weekly and one in the 3-weekly arm, respectively, while one patient was found ineligible after randomization in the weekly arm (Figure 1). Baseline characteristics of the patients are well balanced between the two arms, with the exception of ECOG performance status ≥1 and visceral involvement that were slightly more frequent in the weekly arm (Table 2).

**Table 2:** Description of patients by treatment arm and clinical setting

	Whole set		Locally advanced		Metastatic not pre-treated with anthracyclines		Metastatic pre-treated with anthracyclines	
				
	3-weekly N = 69	Weekly N = 66	3-weekly N = 22	Weekly N = 20	3-weekly N = 26	Weekly N = 26	3-weekly N = 21	Weekly N = 20
Age, median (range), yrs	52 (33-68)	50 (30-69)	48 (33-65)	45 (30-66)	54 (34-67)	53 (38-66)	52 (36-68)	54 (31-69)
ECOG performance status, n (%)								
0	60 (87.0)	49 (74.2)	21 (95.5)	19 (95.0)	21 (80.8)	16 (61.5)	18 (85.7)	14 (70.0)
1	7 (10.1)	15 (22.7)	1 (4.5)	1 (5.0)	4 (15.4)	9 (34.6)	2 (9.5)	5 (25.0)
2	2 (2.9)	2 (3.1)	-	-	1 (3.8)	1 (3.8)	1 (4.8)	1 (5.0)
ER or PgR positive, n (%)	40 (58.0)	38 (57.6)	10 (45.5)	7 (31.8)	17 (65.4)	19 (73.1)	13 (61.9)	12 (60.0)
Previous epirubicin, n (%)	-	-	-	-	-	-	21 (100)	20 (100)
neoadjuvant							3 (14.3)	1 (5.0)
adjuvant							18 (85.7)	17 (85.0)
both							-	2 (10.0)
Dose of previous epirubicin, median (range), mg/m²	-	-	-	-	-	-	422 (340-600)	485 (289-779)
At least one target lesion, n (%)	53 (76.8)	45 (68.2)	22 (100)	17 (77.3)	20 (76.9)	17 (65.4)	11 (52.4)	11 (55.0)
Dominant site of disease, n (%)								
soft tissues (chest wall, breast and nodes)	31 (44.9)	22 (33.3)	22 (100)	20 (100)	6 (23.1)	1 (3.8)	3 (14.3)	1 (5.0)
bone	6 (8.7)	4 (6.1)	-	-	5 (19.2)	2 (7.7)	1 (4.8)	2 (10.0)
viscera (including pleura)	32 (46.4)	40 (60.6)	-	-	15 (57.7)	23 (88.5)	17 (81.0)	17 (85.0)

### Treatment compliance

Median average relative dose intensity of treatment was similar in the two arms; 75% and 70% of patients received all the planned therapy in the control and experimental arms, respectively. The rate of patients receiving all planned cycles of docetaxel and capecitabine was quite low in both the 3-weekly and weekly arms (47.6% and 40.0%, respectively - see Additional File 1).

### Quality of life

Compliance with quality of life assessment was 94% and 70% in the 3-weekly and 97% and 62% in the weekly arm, at baseline and 6 weeks, respectively. There was a statistically significant (p = 0.03) difference in terms of global quality of life scores favouring the control arm (Table 3 and additional file 2a). Among the five functional sub-scores of the EORTC QLQ-C30, patients treated with weekly schedules presented a significantly worsening in role functioning (p = 0.02). Among the nine symptom sub-scores of the QLQ-C30 questionnaire (Additional file 2b), no significant differences were found between 3-weekly and weekly arms, except for the financial sub-score that was worse in the weekly than in the control arm (p < 0.001).

**Table 3:** EORTC quality of life scores by treatment arm

	Mean baseline score (SD)		Mean 6 weeks score (SD)		Mean difference (SD)		
				
	3-weekly	Weekly	3-weekly	Weekly	3-weekly	Weekly	P value*
	N = 48	N = 41	N = 48	N = 41			
**Global QoL**	62.8 (22.8)	64.0 (21.2)	67.9 (16.0)	60.0 (19.0)	5.0 (26.0)	-3.5 (20.5)	**0.03**

**Functional scales**							
Physical functioning	86.3 (16.6)	81.9 (19.6)	82.3 (16.8)	78.0 (18.0)	-4.0 (11.8)	-3.9 (14.3)	0.64
Role functioning	84.7 (22.2)	79.3 (26.0)	84.0 (21.2)	72.0 (23.4)	-0.7 (22.3)	-7.3 (24.7)	**0.02**
Emotional functioning	59.2 (23.5)	60.4 (22.2)	71.5 (19.1)	64.8 (24.4)	12.3 (21.6)	4.5 (18.5)	0.055
Cognitive functioning	88.9 (14.7)	89.8 (21.0)	90.3 (14.9)	83.7 (23.7)	1.4 (19.1)	-6.1 (18.5)	0.055
Social functioning	81.6 (21.5)	80.5 (25.0)	80.2 (26.6)	69.5 (28.6)	-1.4 (32.8)	-11.0 (29.7)	0.07

**Symptoms**							
Pain	23.6 (27.9)	26.0 (25.3)	15.3 (19.1)	22.4 (19.9)	-8.3 (23.8)	-3.7 (19.9)	0.08
Loss of appetite	13.2 (23.6)	13.8 (23.5)	15.3 (21.7)	14.6 (21.1)	2.1 (29.5)	0.8 (24.1)	0.86
Constipation	13.9 (16.6)	15.4 (24.8)	17.7 (23.9)	20.0 (28.0)	4.3 (26.6)	5.8 (31.9)	0.71
Financial	18.8 (26.5)	19.5 (30.7)	15.3 (23.8)	35.0 (33.3)	-3.5 (22.0)	15.4 (37.3)	**0.0007**
Fatigue	19.4 (19.7)	24.1 (25.0)	31.6 (19.0)	36.9 (20.6)	12.2 (16.0)	12.7 (26.1)	0.39
Nausea/vomiting	5.2 (12.0)	7.3 (14.5)	17.4 (25.0)	19.5 (22.6)	12.2 (24.0)	12.2 (21.4)	0.87
Sleeping disturbance	27.7 (28.9)	40.0 (31.3)	22.9 (26.8)	29.2 (32.2)	-5.7 (28.1)	-10.8 (28.6)	0.90
Diarrhoea	5.6 (14.3)	2.6 (9.0)	9.0 (17.9)	12.2 (20.8)	3.5 (19.7)	9.4 (20.2)	0.31
Dyspnoea	5.6 (12.6)	14.6 (25.9)	9.0 (16.5)	19.2 (21.2)	3.5 (15.7)	4.2 (25.2)	0.10

SD: standard deviation; * bold p-values are statistically significant

**Figure 2:**
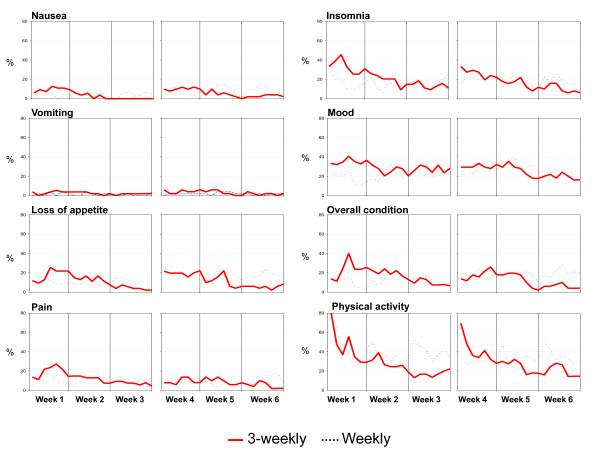
**Pattern of worst scores reported by the Daily Diary Card during the first 6 weeks of treatment**. Gray lines represent weekly schedules of docetaxel; red lines represent 3-weekly schedules of docetaxel.

Daily Diary Cards were compiled and delivered by 55 and 52 patients in the 3-weekly and by 51 and 46 in the weekly arm, after 3 and 6 weeks, respectively. The rates of patients falling into the two worst categories for each item day by day (Figure 2) show that nausea and vomiting had negligible impact in both arms. Daily profiles were consistent with a negative impact of 3-weekly treatment in the first week and the reverse during subsequent weeks.

### Tumor response and overall survival

Both therapeutic regimens showed similar antitumor activity. The response rate was 39.1% (95% exact CI: 27.6-51.6) in the 3-weekly and 33.3% (95% exact CI: 22.2-46.0) in the weekly arm. Seven patients (10%) showed a complete response in the 3-weekly and 2 patients (3%) in the weekly arm.

Median follow-up was 32.9 months in the 3-weekly and 33.0 months in the weekly arm. After 86 (64%) events, the median progression-free survival was 15.2 months in the 3-weekly and 13.1 months in the weekly arm (HR = 1.29; 95% exact CI: 0.84-1.97). With regard to overall survival, 59 events were reported. The median overall survival was 42.9 months in the 3-weekly and 33 months in the weekly arm (HR = 1.38; 95% exact CI: 0.82-2.30). Data on efficacy are summarized in Table 4, where also data scattered by treatment group are reported.

**Table 4:** Activity and efficacy by treatment arm and clinical setting

	Whole set		Locally advanced		Metastatic not pre-treated with anthracyclines		Metastatic pre-treated with anthracyclines	
				
	3-weekly N = 69	Weekly N = 66	3-weekly N = 22	Weekly N = 20	3-weekly N = 26	Weekly N = 26	3-weekly N = 21	Weekly N = 20
**Objective response**								
Complete response, n	7	2	2	1	2	-	3	1
Partial response, n	20	20	9	9	7	7	4	4
Stable disease, n	19	16	10	8	8	7	1	1
Progressive disease, n	5	9	-	1	2	4	3	4
Not evaluable (non responding), n	18	19	1	1	7	8	10	10
Response rate, % (95% exact CI)	39.1 (27.6-51.6)	33.3 (22.2-46.0)	50.0 (28.2-71.8)	50.0 (27.2-72.8)	34.6 (17.2-55.7)	26.9 (11.6-47.8)	33.3 (14.6-57.0)	25.0 (8.7-49.1)
**Progression free survival**								
Progressed patients, n (%)	42 (60.9)	44 (66.7)	6 (27.3)	6 (30.0)	18 (69.2)	20 (76.9)	18 (85.7)	18 (90.0)
Median, months (95% CI)	15.2 (11.8-36.6)	13.1 (7.7-22.4)	n.r.	n.r.	11.9 (10.2-36.6)	10.4 (6.2-22.4)	9.1 (5.9-29.7)	6.2 (4.2-14.4)
HR weekly vs 3 weekly (95% CI)	1.29 (0.84-1.97)		1.10 (0.35-3.45)		1.29 (0.67-2.47)		1.52 (0.78-2.97)	
**Overall survival**								
Dead patients, n (%)	27 (39.1)	32 (48.5)	3 (13.6)	2 (10.0)	13 (50.0)	15 (57.7)	11 (52.4)	15 (75.0)
Median, months (95% CI)	42.9 (27.7-na)	33.0 (25.2-na)	n.r.	n.r.	26.7 (21.2-na)	28.5 (11.4-na)	33.2 (16.3-na)	28.8 (19.8-na)
HR weekly vs 3 weekly (95% CI)	1.38 (0.82-2.30)		0.78 (0.13-4.69)		1.41 (0.67-2.99)		1.54 (0.70-3.36)	

n.r. = not reached

### Toxicity

Hematological and non-hematological toxicities are summarized in Table 5. Among hematological toxicities, neutropenia was common and it was more frequent and severe in the 3-weekly arm, where two patients also experienced febrile neutropenia. Conversely, no relevant differences were observed in anaemia and thrombocytopenia between the two arms. Among non-hematological toxicities, stomatitis was worse in the 3-weekly arm, while no striking differences between the two arms were found in other non-hematological toxicities. Two toxic deaths were observed, both in the 3-weekly arm with the combination of docetaxel and epirubicin: one patient, 67 years old, with metastatic disease, suffered of grade 4 anemia, neutropenia, thrombocytopenia and stomatitis after the third cycle of chemotherapy and died for neutropenic infection; the other patient, 50 years old, with locally advanced disease, died for rectal bleeding nine days after the first cycle of chemotherapy. Data of severe toxicity scattered by arm and treatment group are reported in the Additional file 3.

**Table 5:** Worst NCI-CTC grade of toxicity by patient, in the whole group of patients

	**3-weekly, n (%)**												**Weekly, n (%)**											
		
**Grade**	**0**		**1**		**2**		**3**		**4**		**5**		**0**		**1**		**2**		**3**		**4**		**5**	
Allergy	68	(98,6)		-	1	(1,4)	-		-			-	65	(98,5)		-	1	(1,5)		-		-		-
Anaemia	48	(69,6)	13	(18,8)	7	(10,1)	-		1	(1,4)		-	49	(74,2)	8	(12,1)	7	(10,6)	2	(3,0)		-		-
Neutropenia	21	(30,4)		-	3	(4,3)	19	(27,5)	26	(37,7)		-	36	(54,5)	10	(15,2)	9	(13,6)	7	(10,6)	4	(6,1)		-
Thrombocytopenia	66	(95,7)		-	2	(2,9)		-	1	(1,4)		-	64	(97,0)	1	(1,5)		-	1	(1,5)		-		
Febrile Neutropenia	67	(97,1)					1	(1,4)	1	(1,4)		-	66	(100,0)						-		-		-
Neutropenic infection	59	(85,5)	4	(5,8)	5	(7,2)		-		-	1	(1,4)	61	(92,4)	1	(1,5)	4	(6,1)		-		-		-
Bleeding	66	(95,7)	2	(2,9)		-		-		-	1	(1,4)	64	(97,0)	1	(1,5)		-	1	(1,5)		-		-
Cardiac	61	(88,4)	6	(8,7)	1	(1,4)	1	(1,4)		-		-	59	(89,4)	4	(6,1)	2	(3,0)	1	(1,5)		-		-
Fatigue	43	(62,3)	16	(23,2)	10	(14,5)		-		-		-	41	(62,1)	11	(16,7)	13	(19,7)	1	(1,5)		-		-
Fever	60	(87,0)	4	(5,8)	5	(7,2)		-		-		-	62	(93,9)	2	(3,0)	1	(1,5)	1	(1,5)		-		-
Weight loss	69	(100,0)		-		-		-		-		-	64	(97,0)	1	(1,5)	1	(1,5)		-		-		-
Hair loss	24	(34,8)	1	(1,4)	44	(63,8)							33	(50,0)	8	(12,1)	25	(37,9)						
Cutaneous	60	(87,0)	3	(4,3)	4	(5,8)	1	(1,4)	1	(1,4)			51	(77,3)	8	(12,1)	6	(9,1)	1	(1,5)		-		
Conjunctivitis	66	(95,7)	1	(1,4)	2	(2,9)		-		-		-	61	(92,4)	3	(4,5)	2	(3,0)		-		-		-
Anorexia	67	(97,1)	2	(2,9)		-		-		-			66	(100,0)		-		-		-		-		
Constipation	63	(91,3)	4	(5,8)	2	(2,9)		-		-			63	(95,5)	2	(3,0)	1	(1,5)		-		-		
Diarrhoea	53	(76,8)	9	(13,0)	6	(8,7)	1	(1,4)		-		-	56	(84,8)	6	(9,1)	4	(6,1)		-		-		-
Dysgeusia	67	(97,1)	2	(2,9)		-		-					63	(95,5)	3	(4,5)		-		-				
Nausea	26	(37,7)	25	(36,2)	17	(24,6)	1	(1,4)		-			31	(47,0)	26	(39,4)	8	(12,1)	1	(1,5)		-		
Vomiting	46	(66,7)	14	(20,3)	7	(10,1)	1	(1,4)	1	(1,4)		-	46	(69,7)	14	(21,2)	4	(6,1)	2	(3,0)		-		-
Stomatitis	39	(56,5)	18	(26,1)	8	(11,6)	2	(2,9)	2	(2,9)		-	44	(66,7)	17	(25,8)	4	(6,1)	1	(1,5)		-		-
Abdominal pain	58	(84,1)	5	(7,2)	3	(4,3)	3	(4,3)		-		-	60	(90,9)	4	(6,1)	2	(3,0)		-		-		-
Skeletal pain	64	(92,8)	1	(1,4)	4	(5,8)		-		-		-	66	(100,0)		-		-		-		-		-
Diabetes	69	(100,0)		-		-		-		-		-	63	(95,5)		-	1	(1,5)	1	(1,5)	1	(1,5)		-
Hepatic	64	(92,8)	4	(5,8)	1	(1,4)		-		-		-	57	(86,4)	8	(12,1)	1	(1,5)		-		-		-
Neurologic	62	(89,9)	6	(8,7)		-	1	(1,4)		-		-	59	(89,4)	5	(7,6)		-	1	(1,5)	1	(1,5)	-	
Pulmonary	69	(100,0)		-		-		-		-		-	64	(97,0)	2	(3,0)		-		-		-	-	

## Discussion and Conclusions

This study shows that weekly schedules of docetaxel combined with epirubicin or capecitabine were worse than the standard 3-weekly schedules in terms of global quality of life, role functioning and financial scores, despite the worse hematologic toxicity of the latter, as first-line chemotherapy for locally advanced or metastatic breast cancer. Daily quality of life profiles indicate a positive impact of the weekly treatment only in the first week, while it had a negative impact during subsequent weeks. No major differences in antitumor activity were observed with both schedules.

Two randomized trials compared single-agent docetaxel used either every 3 weeks or weekly in patients with metastatic breast cancer [26,27]. Tabernero et al reported no differences in response rate, but less hematological toxicity in the weekly arm, in a study with 83 randomized patients [27]. Rivera et al, in a study with 118 randomized patients, found a higher response rate in the 3-weekly arm (35.6% vs 20.3%), but patients in this arm experienced more toxicity and there was no difference in progression-free and overall survival [26]. However, none of these two studies included a quality of life assessment. In patients with early breast cancer, Sparano et al. recently reported a randomized phase 3 trial comparing weekly versus 3-weekly docetaxel or paclitaxel after doxorubicin plus cyclophosphamide: a higher 5-year disease-free survival rate in the 3-weekly docetaxel arm (81.2% versus 77.6%) was observed [25].

The quality of life of patients with cancer has been identified as a relevant endpoint in research and clinical practice [28]. The present study is, to our knowledge, the first trial comparing weekly with 3-weekly schedules of docetaxel-based chemotherapy with quality of life as primary endpoint. At 6 weeks, which we chose as the time-point for the primary analysis, there was a slight imbalance in compliance with fewer questionnaires returned in the weekly arm; in principle, this might favour the weekly arm due to a positive selection bias. But, given that our conclusions are in favour of the 3-weekly schedules, we don't believe that this difference is critical. Obviously, comparing treatments with different schedules introduces difficulties in quality of life assessment. The EORTC questionnaire explores patient's feelings during the last week; this might partially affect the observed results, because the time chosen for evaluation corresponded to therapy for the weekly and no therapy for the 3-weekly arm. For this reason we added a daily diary card, to describe as more clearly as possible the changes of quality of life during the whole treatment period. The observed results suggest that the choice of a weekly schedule of docetaxel-based chemotherapy does not improve, but even worsens quality of life of patients, although it is associated with a decrease in haematological toxicity. Our interpretation of this result is that the impact of the reduction of haematological side effects of weekly docetaxel regimens is not enough to counterbalance personal, familiar and social disadvantages associated with more frequent admittance to hospital for weekly administration of antineoplastic drugs. We acknowledge, however, that the impact on familiar and social disadvantages might be affected by regional variation of cancer treatment logistic.

There are several potential limitations of our study. First, the study was stopped prematurely and it is underpowered according to initial plan. Early stopping was independent of treatment effects, but was prompted by the slow accrual rate and the publication of the results of the trial by Sparano et al. [25] that made continuation unethical. However, the sample size of our trial is among the largest reported for studies evaluating quality of life in metastatic breast cancer and the study actually has 80% power to detect a 48% improvement in quality of life, that is an effect of medium size, that might be considered as clinically relevant. Second, a questionable issue is that patients at different stages of disease were eligible for the study; different stages, in fact, may imply worse or better QoL levels; however, our plans were based on the consideration that, once balance is warranted through randomization and stratification, the comparison should not be biased. Similar considerations can be done for the heterogeneity of chemotherapeutic regimens used at the different stages. Third, the evidence of efficacy of some of the treatments schedules chosen for this trial was quite low; however, this limitation might also be interpreted as a value of the present trial that provides some more evidence on treatment schedules proposed as promising and no longer studied, like the combination of weekly docetaxel and capecitabine [16]. Thanks to randomization, our data suggest that this weekly combination is less promising than expected.

The results of the present study should not be generalized to weekly regimens with other drugs, such as paclitaxel, that has shown a superior efficacy compared to paclitaxel every 3 weeks, both in adjuvant and in metastatic setting, with a different toxicity profile [25,29]. Similarly, the results of this study should not be generalized to different subpopulations of breast cancer patients; weekly docetaxel has proven active and extremely well tolerated among elderly breast cancer patients, a population that would be at risk of toxicity with standard 3-weekly scheduling [30,31]. In conclusion, in our study the weekly schedules of docetaxel-based chemotherapy appeared to be inferior to the 3-weekly ones in terms of quality of life, among adult patients with locally advanced or metastatic breast cancer and should not be preferred for clinical practice.

## Competing interests

Honoraria: Alessandro Morabito, Roche; Massimo Di Maio, Roche; Francesco Perrone, Roche.

## Authors' contributions

FN, FP, AdM conceived the study; CG, FP, AdM was involved in study design; FN, AG, FDR, GL, CP, VL, ER, EDM, GDA, RT, MR, GB, MDB carried out the acquisition of data; AM, MCP, MDM performed the quality control of the data; MDM, CG performed the statistical analysis; FN, AM, MCP, FP, AdM were involved in the interpretation of data; FN, AM, MCP, MDM, FP, AdM drafted the manuscript; all authors reviewed and approved the final manuscript.

## Pre-publication history

The pre-publication history for this paper can be accessed here:

http://www.biomedcentral.com/1471-2407/11/75/prepub

## Supplementary Material

Additional file 1 **Table A1**. Treatment compliance by treatment arm and clinical setting. Click here for file

Additional file 2 **Figure A1**. Mean change in EORTC quality of life scores at 6 weeks from baseline (a = functional scales; b = symptom scales). Gray bars represent weekly schedules of docetaxel; red bars represent 3-weekly schedules of docetaxel. Click here for file

Additional file 3 **Table A2**. Grade 3 or worse toxicity (according to NCI-CTC) by treatment arm and clinical setting. Click here for file

## References

[B1] Ravdin PM Burris HA III Cook G Eisenberg P Kane M Bierman WA Mortimer J Genevois E Bellet RE Phase II trial of docetaxel in advanced anthracycline-resistant or anthracenedione-resistant breast cancer J Clin Oncol 1995 13 2879 2885 8523050 10.1200/JCO.1995.13.12.2879

[B2] Valero V Holmes FA Walters RS Theriault RL Esparza L Fraschini G Fonseca GA Bellet RE Buzdar AU Hortobagyi GN Phase II trial of docetaxel: a new highly effective antineoplastic agent in the management of patients with anthracycline-resistant metastatic breast cancer J Clin Oncol 1995 13 2886 2894 8523051 10.1200/JCO.1995.13.12.2886

[B3] Nabholtz JM Senn HJ Bezwoda WR Melnychuk D Deschênes L Douma J Vandenberg TA Rapoport B Rosso R Trillet-Lenoir V Drbal J Molino A Nortier JWR Richel DJ Nagykalnai T Siedlecki P Wilking N Genot JY Hupperets PSGJ Pannuti F Skarlos D Tomiak EM Murawsky M Alakl M Riva A Aapro M 304 Study Group Prospective randomized trial of docetaxel versus mitomycin plus vinblastine in patients with metastatic breast cancer progressing despite previous anthracycline-containing chemotherapy J Clin Oncol 1999 17 1413 1424 304 Study Group 10334526 10.1200/JCO.1999.17.5.1413

[B4] Sjöström J Blomqvist C Mouridsen H Pluzanska A Ottosson-Lönn S Bengtsson NO Ostenstad B Mjaaland I Palm-Sjövall M Wist E Valvere V Anderson H Bergh J Docetaxel compared with sequential methotrexate and 5-fluorouracil in patients with advanced breast cancer after anthracycline failure: A randomised phase III study with crossover on progression by the Scandinavian Breast Group Eur J Cancer 1999 35 1194 1200 10615229 10.1016/s0959-8049(99)00122-7

[B5] Chan S Friedrichs K Noel D Pintér T Van Belle S Vorobiof D Duarte R Gil Gil M Bodrogi I Murray E Yelle L von Minckwitz G Korec S Simmonds P Buzzi F González Mancha R Richardson G Walpole E Ronzoni M Murawsky M Alakl M Riva A Crown J The 303 Study Group Prospective randomized trial of docetaxel versus doxorubicin in patients with metastatic breast cancer J Clin Oncol 1999 17 2341 2354 10561296 10.1200/JCO.1999.17.8.2341

[B6] Nabholtz JM Falkson C Campos D Szanto J Martin M Chan S Pienkowski T Zaluski J Pinter T Krzakowski M Vorobiof D Leonard R Kennedy I Azli N Murawsky M Riva A Pouillart P TAX 306 Study Group Docetaxel and doxorubicin compared with doxorubicin and cyclophosphamide as first-line chemotherapy for metastatic breast cancer: results of a randomized, multicenter, phase III trial J Clin Oncol 2003 21 968 975 10.1200/JCO.2003.04.040 12637459

[B7] Bontenbal M Creemers GJ Braun HJ de Boer AC Janssen JT Leys RB Ruit JB Goey SH van der Velden PC Kerkhofs LG Schothorst KL Schmitz PI Bokma HJ Verweij J Seynaeve C Dutch Community Setting Trial for the Clinical Trial Group Phase II to III study comparing doxorubicin and docetaxel with fluorouracil, doxorubicin, and cyclophosphamide as first-line chemotherapy in patients with metastatic breast cancer: results of a Dutch community setting trial for the clinical trial group of the comprehensive cancer Centre J Clin Oncol 2005 23 7081 7088 10.1200/JCO.2005.06.236 16192591

[B8] Bear HD Anderson S Brown A Smith R Mamounas EP Fisher B Margolese R Theoret H Soran A Wickerham DL Wolmark N The effect on tumor response of adding sequential preoperative docetaxel to preoperative doxorubicin and cyclophosphamide: preliminary results from National Surgical Adjuvant Breast and Bowel Project Protocol B-27 J Clin Oncol 2003 21 4165 4174 10.1200/JCO.2003.12.005 14559892

[B9] Smith IC Heys SD Hutcheon AW Miller ID Payne S Gilbert FJ Ah-See AK Eremin O Walker LG Sarkar TK Eggleton SP Ogston KN Neoadjuvant chemotherapy in breast cancer: significantly enhanced response with docetaxel J Clin Oncol 2002 20 1456 1466 10.1200/JCO.20.6.1456 11896092

[B10] O'Shaughnessy J Miles D Vukelja S Moiseyenko V Ayoub JP Cervantes G Fumoleau P Jones S Lui WY Mauriac L Twelves C Van Hazel G Verma S Leonard R Superior survival with capecitabine plus docetaxel combination therapy in anthracycline-pretreated patients with advanced breast cancer: phase III trial results J Clin Oncol 2002 20 2812 2823 12065558 10.1200/JCO.2002.09.002

[B11] Hainsworth JD Burris HA Erland JB Thomas M Greco FA Phase I trial of docetaxel administered by weekly infusion in patients with advanced refractory cancer J Clin Oncol 1998 16 2164 2168 9626217 10.1200/JCO.1998.16.6.2164

[B12] Tomiak E Piccart MJ Kerger J Lips S Awada A de Valeriola D Ravoet C Lossignol D Sculier JP Auzannet V Phase I study of docetaxel administered as a 1-hour intravenous infusion on a weekly basis J Clin Oncol 1994 12 1458 1467 7912725 10.1200/JCO.1994.12.7.1458

[B13] Burstein HJ Manola J Younger J Parker LM Bunnell CA Scheib R Matulonis UA Garber JE Clarke KD Shulman LN Winer EP Docetaxel administered on a weekly basis for metastatic breast cancer J Clin Oncol 2000 18 1212 1219 10715290 10.1200/JCO.2000.18.6.1212

[B14] Hainsworth JD Burris HA Yardley DA Bradof JE Grimaldi M Kalman LA Sullivan T Baker M Erland JB Greco FA Weekly docetaxel in the treatment of elderly patients with advanced breast cancer: a Minnie Pearl Cancer Research Network phase II trial J Clin Oncol 2001 19 3500 3505 11481356 10.1200/JCO.2001.19.15.3500

[B15] Wenzel C Locker GJ Pluschnig U Zielinski CC Rudas M Oberhuber G Gnant MF Taucher S Jakesz R Steger GG Phase I\II trial of weekly epidoxorubicin and docetaxel (wED) in the neoadjuvant and palliative treatment of patients with breast cancer Cancer Chemother Pharmacol 2002 50 155 159 10.1007/s00280-002-0476-9 12172982

[B16] Nadella P Shapiro C Otterson A Hauger M Erdal S Kraut E Clinton S Shah M Stanek M Monk P Villalona-Calero MA Pharmacologically based scheduling of capecitabine and docetaxel results in antitumor activity in resistant human malignancies J Clin Oncol 2002 20 2616 2623 10.1200/JCO.2002.22.030 12039922

[B17] Sawada N Ishikawa T Fukase Y Nishida M Yoshikubo T Ishitsuka H Induction of thymidine phosphorylase activity and enhancement of capecitabine efficacy by taxol/taxotere in human cancer xenografts Clin Cancer Res 1998 4 1013 1019 9563897

[B18] de Matteis A Nuzzo F D'Aiuto G Labonia V Landi G Rossi E Mastro AA Botti G De Maio E Perrone F Docetaxel plus epidoxorubicin as neoadjuvant treatment in patients with large operable or locally advanced carcinoma of the breast. A single-center phase II study Cancer 2002 94 895 901 10.1002/cncr.20335 11920456

[B19] Trudeau ME Crump M Latreille J Pritchard KI Palmer M Tu D Shepherd L Shear N Shapiro L Oldfield S Escalating Doses of Docetaxel and Epirubicin as First Line Therapy for Metastatic Breast Cancer. A Phase I/II Study of the National Cancer Institute of Canada---Clinical Trials Group Proc Am Soc Clin Oncol 1999 19 abstr 443

[B20] Pronk L Vasey PA Sparreboom A Reigner B Planting AS Gordon RJ Osterwalder B Verweij J Twelves C A phase I and pharmacokinetic study of the combination of capecitabine and docetaxel in patients with advanced solid tumours Br J Cancer 2000 83 22 29 10.1054/bjoc.2000.1160 10883663 PMC2374547

[B21] Aaronson NK Ahmedzai S Bergman B Bullinger M Cull A Duez NJ Filiberti A Flechtner H Fleishman SB de Haes JC Kaasa S Klee M Osoba D Razavi D Rofe PB Schraub S Sneeuw K Sullivan M Takeda F The European Organization for Research and Treatment of Cancer QLQ-C30: a quality of life instrument for use in international clinical trials in oncology J Natl Cancer Inst 1993 85 365 376 10.1093/jnci/85.5.365 8433390

[B22] Fayers P Aaronson NK Bjordal K Curran D Groenveld M EORTC EORTC QLQ-C30 Scoring Manual 1999 2 Brussels (Belgium) 1 77

[B23] Fayers PM Bleehen NM Girling DJ Stephens RJ Assessment of quality of life in small-cell lung cancer using a Daily Diary Card developed by the Medical Research Council Lung Cancer Working Party Br J Cancer 1991 64 299 306 10.1038/bjc.1991.296 1654074 PMC1977498

[B24] Schemper M Smith TL A note on quantifying follow-up in studies of failure time Control Clin Trials 1996 17 343 346 10.1016/0197-2456(96)00075-X 8889347

[B25] Sparano JA Wang M Martino S Jones V Perez EA Saphner T Wolff AC Sledge GW Jr Wood WC Davidson NE Weekly paclitaxel in the adjuvant treatment of breast cancer N Engl J Med 2008 358 1663 1671 10.1056/NEJMoa0707056 18420499 PMC2743943

[B26] Rivera E Mejia JA Arun BK Adinin RB Walters RS Brewster A Broglio KR Yin G Esmaeli B Hortobagyi GN Valero V Phase 3 study comparing the use of docetaxel on an every-3-week versus weekly schedule in the treatment of metastatic breast cancer Cancer 2008 112 1455 1461 10.1002/cncr.23321 18300256

[B27] Tabernero J Climent MA Lluch A Albanell J Vermorken JB Barnadas A Antón A Laurent C Mayordomo JI Estaun N Losa I Guillem V Garcia-Conde J Tisaire JL Baselga J A multicentre, randomised phase II study of weekly or 3-weekly docetaxel in patients with metastatic breast cancer Ann Oncol 2004 15 1358 1362 10.1093/annonc/mdh349 15319242

[B28] Iconomou G Mega V Koutras A Iconomou AV Kalofonos HP Prospective assessment of emotional distress, cognitive function, and quality of life in patients with cancer treated with chemotherapy Cancer 2004 101 404 411 10.1002/cncr.20385 15241840

[B29] Seidman AD Berry D Cirrincione C Harris L Muss H Marcom PK Gipson G Burstein H Lake D Shapiro CL Ungaro P Norton L Winer E Hudis C Randomized phase III trial of weekly compared with every-3-weeks paclitaxel for metastatic breast cancer, with trastuzumab for all HER-2 overexpressors and random assignment to trastuzumab or not in HER-2 nonoverexpressors: final results of Cancer and Leukemia Group B protocol 9840 J Clin Oncol 2008 26 1642 1649 10.1200/JCO.2007.11.6699 18375893

[B30] Hainsworth JD Burris HA III Yardley DA Bradof JE Grimaldi M Kalman LA Sullivan T Baker M Erland JB Greco FA Weekly docetaxel in the treatment of elderly patients with advanced breast cancer: a Minnie Pearl Cancer research network phase II trial J Clin Oncol 2001 19 3500 3505 11481356 10.1200/JCO.2001.19.15.3500

[B31] Nuzzo F Morabito A De Maio E Di Rella F Gravina A Labonia V Landi G Pacilio C Piccirillo MC Rossi E D'Aiuto G Thomas R Gori S Colozza M De Placido S Lauria R Signoriello G Gallo C Perrone F de Matteis A Weekly docetaxel versus CMF as adjuvant chemotherapy for elderly breast cancer patients: safety data from the multicentre phase 3 randomised ELDA trial Crit Rev Oncol Hematol 2008 66 171 80 10.1016/j.critrevonc.2007.10.006 18160303

