# Supplementary material for: Effects on quality of life of weekly docetaxel-based chemotherapy in patients with locally advanced or metastatic breast cancer: results of a single-centre randomized phase 3 trial
Source: BMC Cancer. 2011 Feb 16;11:75. doi: 10.1186/1471-2407-11-75 (PMC3050853; doi:10.1186/1471-2407-11-75)
Supplement: Additional file 1 — Table A1. Treatment compliance by treatment arm and clinical setting. [file 1471-2407-11-75-S1.DOC]

| Table 1A. Treatment compliance by treatment arm and clinical setting | | | | | | | | | |
| --- | --- | --- | --- | --- | --- | --- | --- | --- | --- |
|  |  | **Locally advanced** | |  | **Metastatic not pre-treated with anthracyclines** | |  | **Metastatic pre-treated with anthracyclines** | |
|  |  | **3-weekly**  **N=22** | **Weekly**  **N=20** |  | **3-weekly**  **N=26** | **Weekly**  **N=26** |  | **3-weekly**  **N=21** | **Weekly**  **N=20** |
| Planned number of cycles |  | 4 | 3 |  | 6 | 6 |  | 6 | 6 |
| **Planned duration of each cycle, weeks** |  | 3 | 4 |  | 3 | 4 |  | 3 | 4 |
| **Patients completing planned cycles, n (%)** |  | 21 (95.5) | 19 (95.0) |  | 21 (80.8) | 19 (73.1) |  | 10 (47.6) | 8 (40.0) |
| **Weeks on treatment, median (range)** |  | 13 (3-17) | 13 (9-15) |  | 20 (9-29) | 25 (4-35) |  | 19 (4-23) | 18 (4-31) |
| **Docetaxel, median (range)** |  |  |  |  |  |  |  |  |  |
| total dose, mg/m² |  | 317 (82-326) | 303 (207-417) |  | 448 (114-476) | 521 (28-550) |  | 380 (71-458) | 486 (33-658) |
| dose intensity, mg/m²/week |  | 24.2 (18.8-27.5) | 23.7 (18.0-29.8) |  | 22.1 (9.1-25.6) | 20.3 (7.1-23.2) |  | 17.9 (3.0-22.0) | 19.6 (4.0-31.3) |
| relative dose intensity |  | 0.90 (0.70-1.03) | 0.90 (0.69-1.13) |  | 0.88 (0.37-1.02) | 0.90 (0.32-1.03) |  | 0.92 (0.67-1.05) | 0.86 (0.32-1.01) |
| **Epirubicin, median (range)** |  |  |  |  |  |  |  |  |  |
| total dose, mg/m² |  | 297 (75-304) | 261 (172-333) |  | 359 (91-454) | 421 (23 –510) |  | - | - |
| dose intensity, mg/m²/week |  | 22.8 (17.6-25.7) | 20.3 (16.1-23.8) |  | 17.6 (7.3-24.4) | 16.2 (5.7-22.0) |  | - | - |
| relative dose intensity |  | 0.91 (0.70-1.03) | 0.90 (0.72-1.06) |  | 0.88 (0.37-1.22^1^) | 0.86 (0.30-1.17^2^) |  | - | - |
| **Capecitabine, median (range)** |  |  |  |  |  |  |  |  |  |
| total dose, g/m² |  | - | - |  | - | - |  | 107.2 (2.1-168.0) | 102.8 (0-134.0) |
| dose intensity, g/m²/week |  | - | - |  | - | - |  | 7.6 (0.7-9.6) | 3.7 (0-6.0) |
| relative dose intensity |  | - | - |  | - | - |  | 0.81 (0.08-1.03) | 0.83 (0-1.37^3^) |
| **Need of GCSF, n (%)** |  | n.a.^4^ | 2 (10.0) |  | 11 (42.3) | 3 (11.5) |  | 6 (28.6) | 2 (10.0) |
| ^1,2,3^ Dosing errors: ^1^ 75 mg/m² instead of 60 mg/m²; ^2^ 30 mg/m² instead of 25 mg/m²; ^3^ 1000 mg/m²/bid instead of 625 mg/m²/bid. ^4^ not assessed according to protocol | | | | | | | | | |
