# Supplementary material for: Effects on quality of life of weekly docetaxel-based chemotherapy in patients with locally advanced or metastatic breast cancer: results of a single-centre randomized phase 3 trial
Source: BMC Cancer. 2011 Feb 16;11:75. doi: 10.1186/1471-2407-11-75 (PMC3050853; doi:10.1186/1471-2407-11-75)
Supplement: Additional file 2 — Figure A1. Mean change in EORTC quality of life scores at 6 weeks from baseline (a = functional scales; b = symptom scales). Gray bars represent weekly schedules of docetaxel; red bars represent 3-weekly schedules of docetaxel. [file 1471-2407-11-75-S2.PPT]

## Slide 1
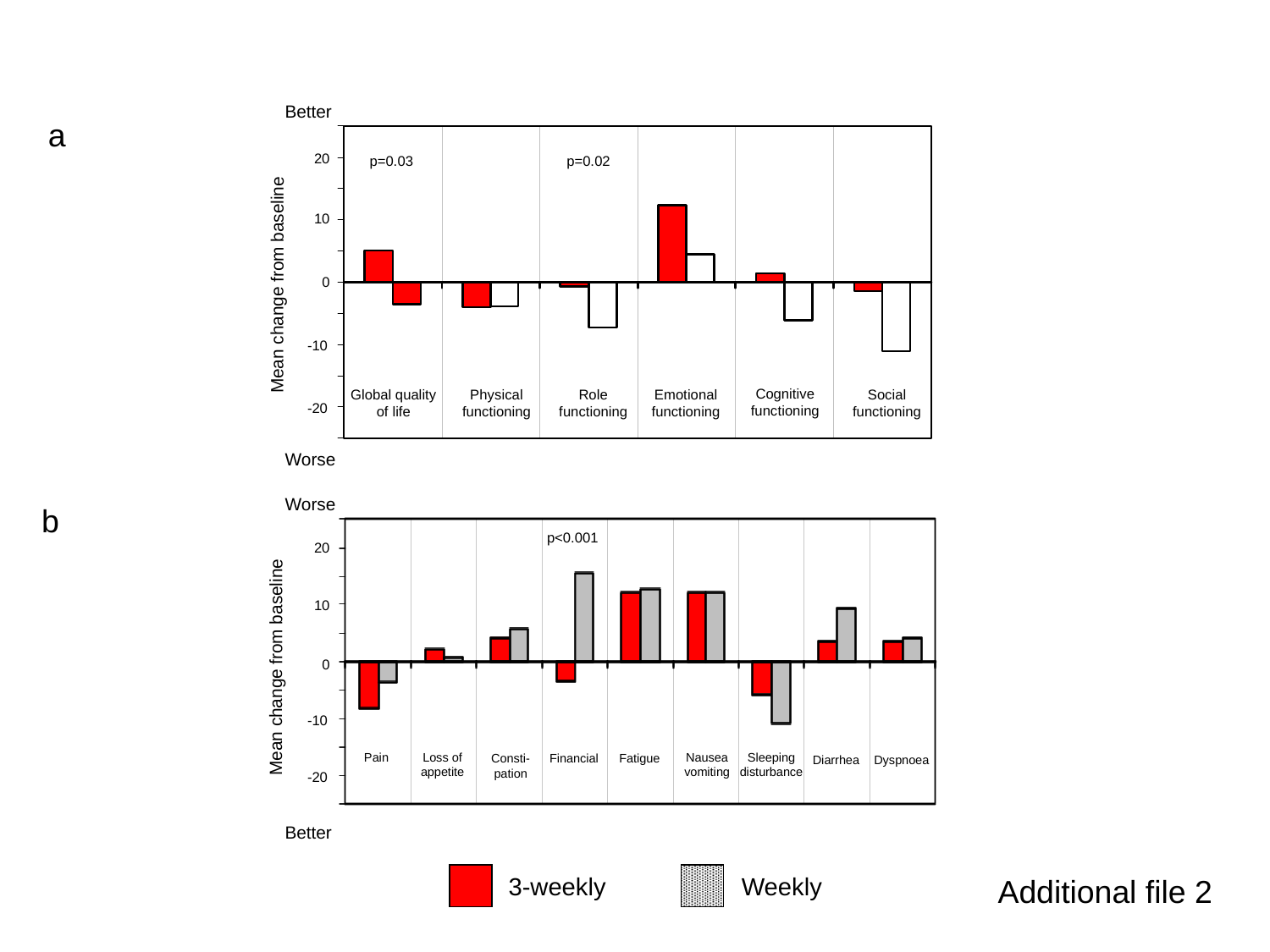

Better
a
20
p=0.03
p=0.02
10
Mean change from baseline
0
-10
Cognitive functioning
Global quality of life
Physical functioning
Role functioning
Emotional functioning
Social functioning
-20
Worse
Worse
b
p<0.001
20
10
Mean change from baseline
0
-10
Pain
Loss of appetite
Nausea vomiting
Sleeping disturbance
Consti-pation
Financial
Fatigue
Diarrhea
Dyspnoea
-20
Better
3-weekly
Weekly
Additional file 2
