# Supplementary material for: Effects on quality of life of weekly docetaxel-based chemotherapy in patients with locally advanced or metastatic breast cancer: results of a single-centre randomized phase 3 trial
Source: BMC Cancer. 2011 Feb 16;11:75. doi: 10.1186/1471-2407-11-75 (PMC3050853; doi:10.1186/1471-2407-11-75)
Supplement: Additional file 3 — Table A2. Grade 3 or worse toxicity (according to NCI-CTC) by treatment arm and clinical setting. [file 1471-2407-11-75-S3.DOC]

| Table 2A. Grade 3 or worse toxicity (according to NCI-CTC) by treatment arm and clinical setting | | | | | | | | | | | | | | | | | | | |
| --- | --- | --- | --- | --- | --- | --- | --- | --- | --- | --- | --- | --- | --- | --- | --- | --- | --- | --- | --- |
|  | **Whole set** | | | |  | **Locally advanced** | | | |  | **Metastatic not pretreated with anthra** | | | |  | **Metastatic pretreated with anthra** | | | |
|  | **3-weekly**  **n=69** | | **weekly**  **n=66** | |  | **3-weekly**  **n=22** | | **weekly**  **n=20** | |  | **3-weekly**  **n=26** | | **weekly**  **n=26** | |  | **3-weekly**  **n=21** | | **weekly**  **n=20** | |
| **Anemia** | 1 | (1,4) | 2 | (3,0) |  | - |  | - |  |  | 1 | (3,8) | 1 | (3,8) |  | - |  | 1 | (5,0) |
| **Neutropenia** | 45 | (65,2) | 11 | (16,7) |  | 15 | (68,2) | 3 | (15,0) |  | 17 | (65,4) | 6 | (23,1) |  | 13 | (61,9) | 2 | (10,0) |
| **Thrombocytopenia** | 1 | (1,4) | 1 | (1,5) |  | - |  | - |  |  | 1 | (3,8) | 1 | (3,8) |  | - |  | - |  |
| **Febrile neutropenia** | 2 | (2,9) | - |  |  | 2 | (9,1) | - |  |  | - |  | - |  |  | - |  | - |  |
| **Neutropenic infection** | 1 | (1,4) | - |  |  | - |  | - |  |  | 1 | (3,8) | - |  |  | - |  | - |  |
| **Bleeding** | 1 | (1,4) | 1 | (1,5) |  | 1 | (4,5) | - |  |  | - |  | 1 | (3,8) |  | - |  | - |  |
| **Cardiac** | 1 | (1,4) | 1 | (1,5) |  | - |  | - |  |  | - |  | - |  |  | 1 | (4,8) | 1 | (5,0) |
| **Fatigue** | - |  | 1 | (1,5) |  | - |  | - |  |  | - |  | - |  |  | - |  | 1 | (5,0) |
| **Fever** | - |  | 1 | (1,5) |  | - |  | - |  |  | - |  | 1 | (3,8) |  | - |  | - |  |
| **Cutaneous** | 2 | (2,9) | 1 | (1,5) |  | - |  | - |  |  | - |  | - |  |  | 2 | (9,5) | 1 | (5,0) |
| **Diarrhoea** | 1 | (1,4) | - |  |  | - |  | - |  |  | - |  | - |  |  | 1 | (4,8) | - |  |
| **Nausea** | 1 | (1,4) | 1 | (1,5) |  | 1 | (4,5) | - |  |  | - |  | - |  |  | - |  | 1 | (5,0) |
| **Vomiting** | 2 | (2,9) | 2 | (3,0) |  | 1 | (4,5) | 1 | (5,0) |  | - |  | - |  |  | 1 | (4,8) | 1 | (5,0) |
| **Stomatitis** | 4 | (5,8) | 1 | (1,5) |  | - |  | - |  |  | 1 | (3,8) | 1 | (3,8) |  | 3 | (14,3) | 1 | (5,0) |
| **Abdominal pain** | 3 | (4,3) | - |  |  | 1 | (4,5) | - |  |  | - |  | - |  |  | 2 | (9,5) | - |  |
| **Diabetes** | - |  | 2 | (3,0) |  | - |  | - |  |  | - |  | - |  |  | - |  | 2 | (10,0) |
| **Neurologic** | 1 | (1,4) | 2 | (3,0) |  | - |  | 1 | (5,0) |  | - |  | - |  |  | 1 | (4,8) | 1 | (5,0) |
| **Toxic death** | 2 | (2,9) | - |  |  | 1 | (4,5) | - |  |  | 1 | (3,8) | - |  |  | - |  | - |  |
